# Impact of Darker, Intermediate and Lighter Phenotypes of Body Melanization on Desiccation Resistance in *Drosophila melanogaster*

**DOI:** 10.1673/031.009.4901

**Published:** 2009-07-08

**Authors:** Ravi Parkash, Subhash Rajpurohit, Seema Ramniwas

**Affiliations:** Department of Biochemistry and Genetics, Maharshi Dayanand University, Rohtak |2400|, India; ^b^ Current address: School of Life Sciences, University of Nevada, 4505 Maryland Parkway, Las Vegas, NV 89154-4004, USA

**Keywords:** abdominal melanization, assorted phenotypes, rate of water loss, climatic adaptation

## Abstract

A possible link between melanization and desiccation resistance can be inferred if within population differences in melanization find significant correlations with desiccation resistance and its mechanistic basis i.e. rate of water loss/hr. Accordingly, darker, intermediate and lighter phenotypes of body melanization were analyzed in wild and laboratory reared *Drosophila melanogaster* L. (Diptera: Clyclorrapha) populations from highland and lowland sites located in close proximity at five different latitudinal locations (11.15 °N to 31.06°N) within the Indian subcontinent. In large population samples, occurrence of significant within population variability made it possible to assort non-overlapping phenotypes of body coloration (i.e. lighter (< 25%), intermediate (30 to 40%) and darker (> 45%)) for all the populations which were further investigated for desiccation resistance and rate of water loss/hr. Significantly, higher desiccation resistance but much reduced rate of water loss/hr were observed in darker and intermediate phenotypes in all the populations. By contrast, lighter phenotypes exhibited lower desiccation tolerance but higher rate of water loss/hr. A regression analysis between traits provided similar slope values for wild and laboratory populations. For all three physiological traits, predicted trait values from multiple regression analysis as a simultaneous function of annual average temperature and relative humidity, matched the observed values. We infer that parallel changes in melanization and desiccation resistance may result from decreasing annual average temperature and relative humidity along increasing latitude as well as altitude on the Indian subcontinent.

## Introduction

In ectothermic insects, melanin patterns are involved in diverse aspects of their ecology (Majerus 1998; Willmer *et al*. 2005). Phenotypic manifestations of melanization differ greatly across various insect taxa. Insect orders such as Lepidoptera and Coleoptera exhibit melanic and non-melanic morphs while some Drosophilids are characterized by continuously varying patterns of body melanization (Wittkopp *et al*. 2003). Both field and laboratory experiments have suggested diverse selection mechanisms in different insect taxa (True 2003). Several investigations favor a role of body melanization in thermoregulation (Watt 1968; de Jong *et al*. 1996). Direct evidence of differential effects of solar radiation on dark vs lightly colored individuals have been obtained for wild and laboratory populations of beetles and butterflies (Brakefield and Willmer 1985; Ellers and Boggs 2004). Fitness consequences of thermoregulation have been found to differ among several insect taxa (Majerus 1998). In butterflies, wing melanism has been associated with longer flight distances (Guppy 1986). However, melanics of the two-spot ladybird beetle, *Adalia bipunctata* are favored in industrial areas where higher smoke pollution has resulted in lower ambient sunlight levels (de Jong and Brakefield 1998). Thus, cuticular melanization is an important interface between physiological systems and the environmental conditions that are complex and include temperature, humidity and other climatic factors (Mani 1968; Neville 1975; Lee and Denlinger 1991). On the Indian subcontinent, average annual temperature (Tave) and relative humidity (RH) decrease along increasing latitude as well as altitude. Thus, ectothermic insect populations face colder and drier conditions from south to north and also with increasing elevation all along the Indian subcontinent. It is not known whether changes in body melanization confer desiccation resistance under drier conditions. A darker body mutant strain (ebony) of *Drosophila* showed higher desiccation resistance but a possible link between melanization and desiccation resistance has not been investigated in wild populations of various insect taxa (Kalmus 1941).

There have been several studies in insects on the rate of water loss in laboratory-selected strains for desiccation resistance but such analyses have not been considered in context of body melanization (Hoffmann and Parsons 1993; Gibbs et al. 1997). the mechanistic basis of desiccation resistance may include reduction in cuticular permeability due to melanization (Hadley 1994). A possible link between melanization and desiccation can be shown if laboratory selected strains for higher melanization show an increase in desiccation resistance, and if within population differences in assorted groups (darker, intermediate and lighter phenotypes) of body melanization show significant parallel changes in desiccation resistance and its mechanistic basis i.e. rate of water loss/hr. In the present investigation, the latter approach was followed and both wild and laboratory populations of *Drosophila melanogaster* L. (Diptera: Clyclorrapha) from diverse geographical locations showed significant associations between darker phenotypes and higher desiccation resistance while lighter phenotypes showed lesser desiccation resistance. Significant reduction in rate of water loss/hr in darker phenotypes could be indirect evidence in favor of cuticular impermeability due to higher body melanization. It is often assumed that phenotypic variability implies adaptation to local environmental conditions. Improved prediction of all the three physiological trait values was obtained using Tave and RH in a multiple regression analysis.

## Materilas and Methods

### Collections

**Figure 1.**
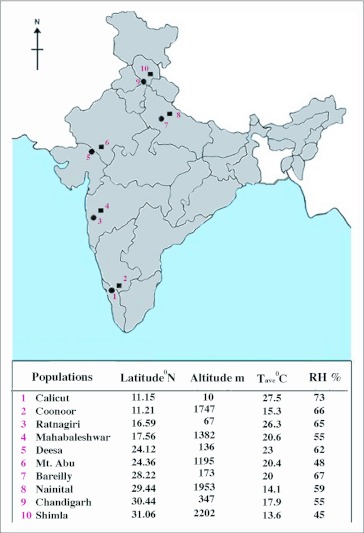
Outline map of India showing collection localities along with their latitude and altitude of origin of ten populations of *Drosophila melanogaster*. For each population, climatic data (Tave °C; RH %) are also given. Filled circles Indicate lowland sites whereas dark rectangles represent highland localities.

Four mass cultures (n = 50 each) per population were set up from field collections in November 2005. From each collection site, about 450 wild-caught individuals were obtained using banana bait traps and net sweeping. The flies were collected from low and high altitudinal sites located in close proximity at each of five latitudinal locations on the Indian sub-continent (Figure 1). The sampling sites were characterized by their latitude, altitude, average annual temperature (Tave) and relative humidity (RH) on the basis of climatological tables (Meteorological Department of India). Wild populations are adapted to different climatic conditions but between population differences can be analyzed on the basis of common garden experiments. For such experiments, flies of each population were reared in oxygen demand incubators at a constant temperature (21 ±0.2°C) on cornmeal- sugar medium for 3 generations before being used for experiments. For all the cultures, density was kept low by limiting oviposition to 6–8 hrs. resulting in about 180–200 flies per stock bottle. The mass cultures were maintained as 2 to 3 replicates. All experiments were performed with G1 to G3 generations on 7–8 day old adults in order to test repeatability of trait values. For each population, progeny from four stock bottles were pooled and sexes were separated. The resulting female individuals were assorted into three groups of darker, intermediate and lighter phenotypes under a stereo microscope (Olympus, www.olympus.com). Six wild populations and ten laboratory populations were analyzed for % body melanization, desiccation resistance and rate of water loss/hr.

### Physiological traits

Body melanization was estimated from a lateral aspect of the female abdomen giving values ranging from 0 (no melanization ) to 10 (complete melanization) for each of the six visible abdominal segments (2^nd^ to 7^th^). Abdominal melanization scores were weighted to the relative sizes of the respective segments. Data on percent melanization were calculated as (Σ observed weighted melanization scores of six abdominal segments per fly/Σ relative size of each abdominal tergite × 10 per fly) × 100. The total body melanization per fly was also estimated through Biowizard image analysis Software. For this purpose only the abdomen of each fly minus viscera was mounted on a slide. For wild as well as laboratory populations, large samples of about 250 females were assorted into three phenotypic classes i.e. darker (> 45%), intermediate (30 to 40%) and lighter (< 25%) phenotypes.

To measure desiccation resistance at 21°C, six replicates of ten female individuals of each phenotype (darker or intermediate or lighter phenotypes) were isolated in dry plastic vials (Parkash and Munjal 1999). These vials contained 2 g of silica gel (a desiccant to maintain relative humidity at ∼5%) at the bottom of each vial and were covered with a disc of plastic foam. These vials were inspected every hour and the number of dead flies (completely immobile) was recorded. When the number of dead individuals approached one half, vials were inspected every 30 minutes till all the flies died. The rate of water loss in live flies due to desiccation for six hours was done in six groups of ten flies per population. Repeatability of this assay was ensured before analyzing populations. Both before and after desiccation, groups of ten flies were weighed on a Sartorius (www.sartorius.com) microbalance and the rate of water loss/hr was calculated.

**Table 1.**
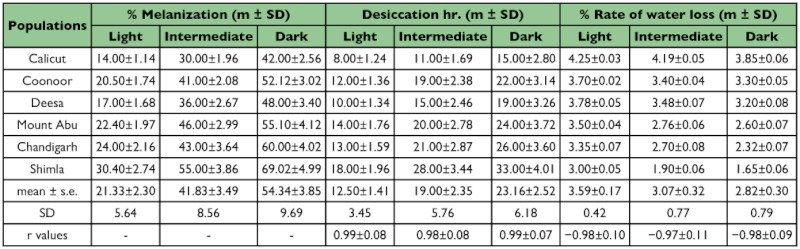
Data on mean ±s.d. of percent body melanisation, desiccation hours and rate of water loss/hr in wild caught assorted groups of darker, Intermediate and lighter phenotypes of six populations of *Drosophila melanogaster*.

### Statistical analyses

For all the three physiological traits, data are given as mean ± SD of female individuals (30 wild caught or 60 laboratory reared) of either darker or intermediate or lighter phenotypes per population. For each trait across populations, means and standard deviation are given for purpose of comparison. Simple regression analysis of relationships between melanization and desiccation resistance, and between melanization and rate of water loss/ hr, were made by comparing slope values for wild and laboratory populations (Zar 1996). For all the three traits, the associations of climatic (Tave and RH) and geographic variables (latitude and altitude) were tested using multiple regression analysis. Statistica (StatSoft Inc., Release 5.0, www.statsoft.com) was used for calculations as well as illustrations.

## Results

### Trait variability

Data for body melanization, desiccation and rate of water loss/hr for wild caught female individuals assorted as darker, intermediate and lighter phenotypes in six populations of *D. melanogaster* are given in Table 1. As shown in the map (Figure 1), paired populations have their origin in southern, central and northern localities that vary significantly in their climatic conditions. Laboratory populations provided larger samples for analysis and each value is an average of 60 individuals as compared with wild caught (n = 30 for each phenotypic category of body melanization ). In the laboratory, trait values showed higher repeatability (> 0.92) across generations. For each wild caught population, three types of melanic phenotypes exhibited significant differences on the basis of ‘t’ test (p< 0.01). On average, darker phenotypes differ from lighter phenotypes by about 30% while intermediate phenotypes had about 5% higher melanization than expected average values. For each population, the variability within each phenotypic category can be appreciated by confidence limits i.e. ± 1.96 SD. Such analysis provided ample variability within each phenotypic category. For samples collected from the wild, each phenotypic category showed parallel changes in desiccation tolerance and rate of water loss/hr and the respective correlation values are highly significant (Table 1). Darker phenotypes always showed higher desiccation resistance and reduced rate of water loss/hr. However, values were lower for desiccation resistance and higher rate of water loss in case of lighter phenotypes. Phenotypic trait variability (SD) for all the three physiological traits was significantly higher for the darker and intermediate phenotypes as compared with lighter phenotypes. When data from six wild caught populations were compared with laboratory populations parallel results were obtained (Table 2). Thus, analysis of laboratory populations further supported the conclusions derived from wild caught samples. Phenotypic trait variability (standard deviation) was significantly higher for wild caught populations as compared with laboratory populations.

### Associations between physiological traits

**Figure 2.**
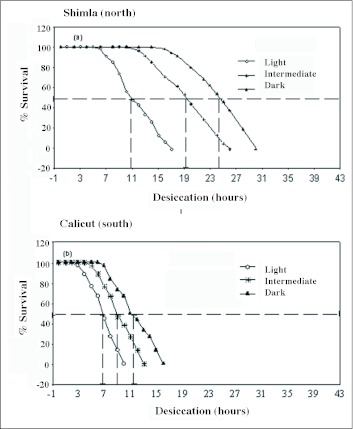
Contrasting patterns of survival curves under desiccating conditions for assorted groups of darker, Intermediate and lighter phenotypes of a northern (a) Shimla and a southern (Calicut) population (b) of Droso*phila melanogaster*.

**Figure 3.**
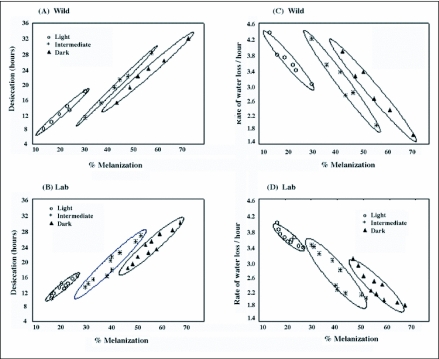
Positive correlation between percent melanization and desiccation hours (a & b) and negative correlation between body melanization and rate of water loss/hr (c & d) for assorted groups of darker, Intermediate and lighter phenotypes in wild and laboratory populations of *Drosophila melanogaster*. Ellipses of 80% probability are shown.

Changes of body melanization across a latitudinal gradient (11.15 to 31.06 °N) form regular clines with significantly lower slope value (b= 0.29) for lighter phenotypes as compared with darker and intermediate phenotypes (b = 0.57 and 0.59 respectively). Such an observation may imply selective disadvantage for lighter phenotypes when moving from southern to northern localities that involve a change toward drier habitats. For each population, the three assorted phenotypic categories of body melanization resulted in significant differences in their desiccation survival curves. Such data have been illustrated only for one northern and one southern population of *D. melanogaster* (Figure 2a and b). A simultaneous analysis of physiological traits on three assorted categories of body melanization provided parallel changes on the basis of significant phenotypic correlations both within as well as between populations. Simple regression analysis helped in comparing slope values (for all the three phenotypic categories) for melanization with desiccation as well as rate of water loss/hr (Table 3). For all phenotypic categories, the slope values were quite similar for wild as well as laboratory populations and in all cases the coefficient of determination (R^2^) was highly significant (R^2^ = 0.78 to 0.98). Thus, for all phenotypic categories the changes in body melanization find positive association with changes in desiccation resistance. However, there is a negative association between body melanization and rate of water loss/hr. These results have been illustrated both for wild caught populations (Figure 3a and b) and for laboratory populations (Figure 3c and d). Although, only six wild populations were investigated as compared with ten laboratory populations, there is higher trait variability for wild caught samples.

**Table 2.**
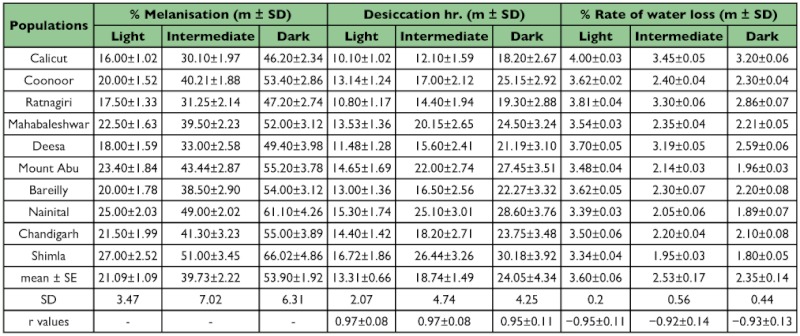
Data on ten laboratory populations (grown at 21°C) of *Drosophila melanogaster* analyzed for three physiological traits in three assorted categories of body melanisation (darker, Intermediate and lighter).

### Analysis with climatic variables

The investigated populations of *D. melanogaster* differ significantly in their latitude as well as altitude of origin as shown in Figure 1. In order to analyze possible effects of local climatic conditions, we considered simultaneously either two geographical parameters or two climatic factors (i.e. average annual temperature; T_ave_ and relative humidity on a yearly basis; RH). For ten localities, latitude is not significantly correlated with altitude as well as with climatic parameters (Tave + RH). However, altitude and T_ave_ were significantly correlated (r = - 0.84 ± 0.19). For geographical parameters, we obtained highly significant values of correlation coefficients for all the physiological traits (Table 4). Altitudinal correlation values were higher when compared for latitude. However, correlations were positive for melanization and desiccation while negative for rate of water loss/hr (Table 4). Two climatic parameters (T_ave_ + RH) are also not significantly correlated for the investigated localities. The rate of water loss has shown significant positive correlation with both T_ave_ and RH while for body melanization and desiccation resistance both climatic variables are negatively correlated (Table 4).

**Figure 4.**
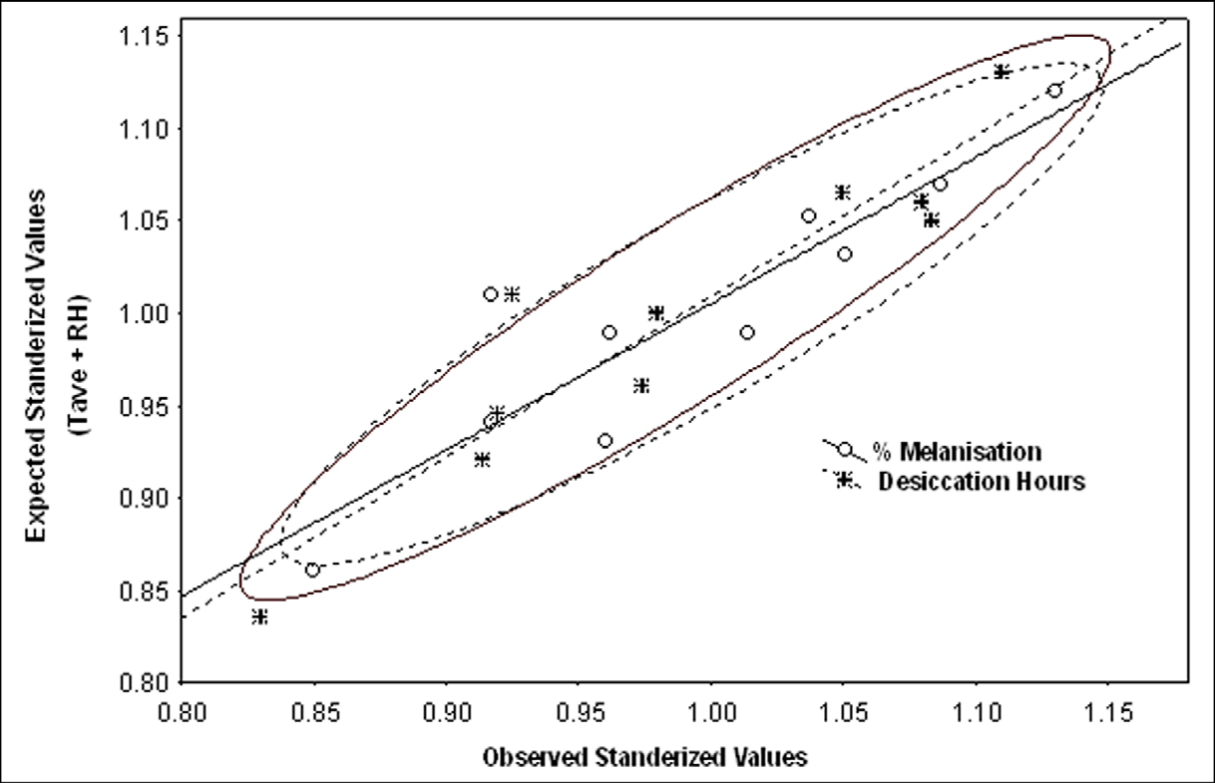
Concordance between expected values of percent melanization and desiccation resistance (on the basis of simultaneous function of Tave and RH of origin of populations) with observed values. The data were standardized for avoiding the scaling effects of both the traits.

We considered multiple regression analysis with geographical parameters and the data are shown in Table 5. For all the three physiological traits, we obtained significantly high values of coefficient of determination (R^2^= 0.92 to 0.99) when both geographical parameters were considered together. Regression values (intercept, slope b1 and b_2_) were used to calculate expected trait value for each population and we found similarity between observed and expected values (data not shown). We attempted multiple regression analysis by considering Tave and RH of the site of origin of populations and found a significant relationship between observed and expected trait values. Figure 4 illustrates a concordance between observed trait values for body melanization as well as desiccation and values expected on the basis of multiple regression analysis of climatic parameters. The data were standardized in order to avoid scaling effects because these two physiological traits involve different scales of measurements. Such analysis favors the view that observed changes in these physiological traits might involve effects of climatic factors (which characterize geographical parameters of origin of populations).

**Table 3.**
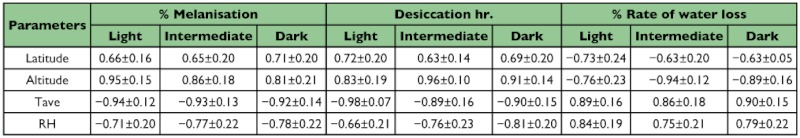
Correlation coefficients between physiological traits and geographical as well as climatic variables on the Indian subcontinent.

**Table 4.**
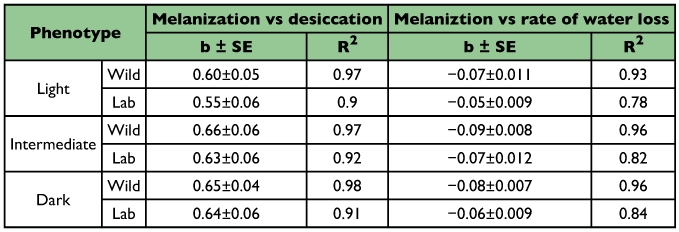
Simple regression analysis of covariation between body melanisation and desiccation resistance; and with rate of water loss/hr in assorted phenotypic groups of wild and laboratory populations of *Drosophila melanogaster*.

## Discussion

Indian subcontinent (8–33 °N) provides a large geographical range covering localities with diverse climatic conditions that favor adaptations of specific phenotypic variations. Southern and northern localities fall under tropical and subtropical climatic conditions because tropic of cancer passes almost midway of the subcontinent. Latitudinally, southern peninsular localities face more humid conditions while northern localities face drier conditions due to seasonal variations. By contrast, higher altitudinal localities, all along the subcontinent, are characterized by much lower temperature and also lower humidities (Mani 1968). Thus, *D. melanogaster* populations face changes in temperature and humidity that vary spatially. The variable climatic conditions on the Indian subcontinent may cause rapid phenotypic changes in traits related to water stress and thermal stress and such expectations match the observed data on three physiological traits. The trait values demonstrate about two fold increase and such changes for all the traits form clines along latitude. All the populations showed significant within population variability i.e. the traits showed 2 to 4 % standard deviation in the investigated populations. Furthermore, between populations variability was significantly higher i.e. about 5 to 8 % standard deviation for wild populations. Thus, for all the three physiological traits, there are parallel changes in trait variability across populations.

**Table 5.**
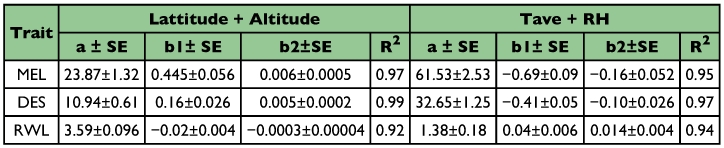
Multiple regression analysis applied to three physiological traits as a simultaneous function of either geographical parameters or/and climatic variables. Pooled data for all the laboratory populations were considered.

Several problems arise with any attempt to find possible causal factors for observed differences in a physiological trait. *D. melanogaster* populations in the Indian subcontinent do face desiccation stress and have evolved desiccation resistance in wild populations along latitude as well as altitude. The well known adaptive mechanisms for desiccation resistance include reduction in rate of water loss through changes in cuticular lipids in diverse insect groups (tenebrionid beetles, grasshoppers and xeric *Drosophila* species) which inhabit hot and dry habitats characteristic of deserts (Gibbs 1998; Gibbs and Matzkin 2001; Zachariassen 1996). By contrast, for the investigated localities, altitudinal populations face lower temperatures as well as lower humidity's that always covary. Our preliminary experiments showed no changes in the amount of cuticular lipids in lowland vs. highland populations (unpublished data). For *D. melanogaster*, ebony mutant flies are known to provide higher desiccation (Kalmus 1941). Accordingly, we analyzed assorted groups of darker, intermediate and lighter flies for wild and laboratory populations which evidenced significant covariation between melanization and desiccation resistance (Table 1 and 2; Figure 3a and b). Darker phenotype evidenced higher desiccation while lighter phenotype showed lower desiccation. Cuticular impermeability due to body melanization can be inferred on the basis of reduced rate of water loss in darker flies. The observed changes in three physiological traits suggest possible link between body melanization and desiccation resistance.

Quantitative traits (body melanization and desiccation resistance) are controlled by polygenes and vary due to interactions of environmental factors with genetic attributes (Falconer and Mackay 1996). Observed variations in the investigated physiological traits show regular clines along latitude as well as altitude. The occurrence of latitudinal clines for various traits (morphological, cytological and ecophysiological) on different continents is often considered to be adaptive evolutionary responses to climatic conditions of the origin of populations (Dobzhansky 1948; Stalker and Carson 1948; Mettler et al. 1977; Prevosti et al. 1985; Watada 1986; Parkash and Munjal 1999; Parkash et al. 2005). However, climatic conditions are complex and it can be difficult to identify environmental selective factors. The explanation for all latitudinal clines is that temperature is responsible for the selection because latitude and temperature are strongly correlated (Hoffmann and Weeks 2007). Unlike temperature, relative humidity shows no consistent pattern in relation to latitude on different continents. Thus, under natural conditions, changes in relative humidity have not been considered as a selective agent. By contrast, on the Indian subcontinent, humidity changes are significant and should be relevant for several traits including desiccation resistance. We followed multiple regression for analyzing trait variability as a simultaneous function of either geographical parameters (latitude and altitude) or climatic variables (Tave and relative humidity) of the sites of origin of populations. If such an analysis provides better prediction of trait variability in terms of significant values of coefficient of determination (R2), we can infer a possible role in the adaptive evolutionary response. We obtained improved R2 values by combining latitudinal and altitudinal effects. Both latitude and altitude itself are not selective factors but are related to climatic selection. For all the physiological traits, average temperature and relative humidity provided significant improvement in the coefficient of determination (R2) and thus, it can be inferred that both could be possible selective factors.

Changes in body melanization may follow quite different consequences in diverse insect taxa (Majerus 1998). The adaptive significance of wing melanization in beetles and moths has been explained on the basis of thermoregulation, Batesian and Mullerian mimicry, aposematism and crypsis (True 2003). By contrast, most *Drosophila* species are of small size (2–3mm) and do not display wing melanization and the role of differential predation may be insignificant as compared with large sized beetles and moths. Furthermore, as compared with diverse types of food resources of beetles and moths, which may influence melanization, *D. melanogaster* mainly feeds on fermented organic matter (i.e. alcoholic substances) that may not produce changes in body melanin. In the present studies, we have not considered these aspects and future investigations are needed to verify such possibilities.

In conclusion, analysis of populations of *D. melanogaster* demonstrates that body melanization is phenotypically variable and subject to natural selection pressure for increased melanization under colder and drier conditions. Our data are consistent with the increased melanization being a consequence of selection on desiccation. Body melanization may result in cuticular impermeability for reducing water loss under increasing dehydrating conditions. The analysis of climatic factors has shown that average temperature (Tave) and relative humidity (RH) might affect phenotypic variability in traits related to thermal stress and resulting changes in water balance requirements.
